# Socio-economic inequity in HIV testing in Malawi

**DOI:** 10.3402/gha.v9.31730

**Published:** 2016-10-26

**Authors:** Sung Wook Kim, Jolene Skordis-Worrall, Hassan Haghparast-Bidgoli, Anni-Maria Pulkki-Brännström

**Affiliations:** 1 Warwick Clinical Trials Unit, Warwick Medical School, University of Warwick, Coventry, United Kingdom; 2 UCL Institute for Global Health, UCL, London, United Kingdom; 3 Epidemiology and Global Health, Department of Public Health and Clinical Medicine, Umeå University, Umeå, Sweden

**Keywords:** inequity, decomposition analysis, HIV testing, socio-economic status, Malawi

## Abstract

**Background:**

Human immunodeficiency virus (HIV) is a significant contributor to Malawi's burden of disease. Despite a number of studies describing socio-economic differences in HIV prevalence, there is a paucity of evidence on socio-economic inequity in HIV testing in Malawi.

**Objective:**

To assess horizontal inequity (HI) in HIV testing in Malawi.

**Design:**

Data from the Demographic and Health Surveys (DHSs) 2004 and 2010 in Malawi are used for the analysis. The sample size for DHS 2004 was 14,571 (women =11,362 and men=3,209), and for DHS 2010 it was 29,830 (women=22,716 and men=7,114). The concentration index is used to quantify the amount of socio-economic-related inequality in HIV testing. The inequality is a primary method in this study. Corrected need, a further adjustment of the standard decomposition index, was calculated. Standard HI was compared with corrected need-adjusted inequity. Variables used to measure health need include symptoms of sexually transmitted infections. Non-need variables include wealth, education, literacy and marital status.

**Results:**

Between 2004 and 2010, the proportion of the population ever tested for HIV increased from 15 to 75% among women and from 16 to 54% among men. The need for HIV testing among men was concentrated among the relatively wealthy in 2004, but the need was more equitably distributed in 2010. Standard HI was 0.152 in 2004 and 0.008 in 2010 among women, and 0.186 in 2004 and 0.04 in 2010 among men. Rural–urban inequity also fell in this period, but HIV testing remained pro-rich among rural men (HI 0.041). The main social contributors to inequity in HIV testing were wealth in 2004 and education in 2010.

**Conclusions:**

Inequity in HIV testing in Malawi decreased between 2004 and 2010. This may be due to the increased support to HIV testing by global donors over this period.

## Introduction

Overall, Malawi has a high-level human immunodeficiency virus (HIV) epidemic. An estimated 1,100,000 people, or approximately 11% of the total population, were living with HIV in 2012 (1). Malawi's HIV prevalence is similar to that of other countries in the southern and eastern regions of sub-Saharan Africa, including Botswana and South Africa (2).

In 2013, the Joint United Nations Programme on HIV/AIDS (UNAIDS) set the ‘90–90–90 goals’ to mobilise the global response to HIV. According to these goals, by 2020, 90% of people living with HIV should be aware of their HIV status, 90% of those known to be HIV positive should be on treatment and 90% of people on treatment should be virally suppressed (3). Malawi is one of five countries in which less than 1 in 10 HIV-exposed children obtained early infant diagnostic services along with Angola, Chad, the Democratic Republic of Congo and Nigeria which are among 21 priority countries (3). In 2014, only 40% of men aged 15–49 years in Malawi had received an HIV test in the previous 12 months despite the generalised nature of the epidemic (4). A better understanding of testing and diagnosis in the Malawian context is critical to the achievement of the 90–90–90 goals. Without access to testing and diagnosis, treatment cannot follow.

Receipt of an HIV test in Malawi is likely to be determined by both need and non-need factors. Gravelle et al. (5) discussed the definition of health equity as ‘equal treatment for equal need’, according to which, *need variables* should affect the use of health service and *non-need variables* should not. Need variables thus reflect health status, while non-need variables tend to reflect socio-economic status (SES) such as wealth and education. Key findings from the Demographic and Health Survey (DHS) 2010 showed that HIV prevalence in Malawi is three times higher for men in the highest income group than for men in the lowest income group (6). HIV prevalence among urban residents is also greater than that in rural areas. For example, urban men are almost twice as likely to be infected as rural men. A similar pattern is observed among women; 11.2% of women living in urban areas are HIV positive, compared with 3.7% living in rural areas (6). This suggests that the need for HIV testing may not be equally distributed across the population in Malawi. However, these figures should be interpreted with caution for at least two reasons. Firstly, the calculation of prevalence rates may be affected by the intensity of testing for HIV. Secondly, although rural prevalence may be lower, absolute numbers of people living with HIV may be greater in rural areas where the majority of the Malawian population resides.

Although a number of studies demonstrate that HIV testing uptake varies by socio-demographic and economic characteristics (2, 7–10), there is a lack of evidence about whether there is equal access for equal need in Malawi. In general, equal treatment for equal need is referred to as horizontal equity (11). ‘Equal access for equal need’ means that patients who have an equal need for a health service make equal use of care without being disproportionately affected by non-need factors such as SES (11). Furthermore, there is little evidence regarding rural–urban differences in HIV testing in Malawi, despite the fact that urbanity is one of the major socio-economic factors widely employed in inequity studies (12–14).

Most studies of access to HIV testing have taken either an urban or a rural focus and have tended to focus on single or clustered districts (15, 16). These study designs preclude urban–rural comparisons and analyses of geographic variation at the national level. For example, Yoder et al. (15) carried out qualitative research on access to HIV testing in Malawi using data from four study sites in Blantyre, Chiradzulu, Lilongwe and Dowa districts. They explored the reasons for why people in those sites sought an HIV test and found that most women receiving an HIV test were worried about HIV infection from their partners. Helleringer et al. (16) studied the uptake of home-based testing in rural areas, including six villages of Likoma Island, and found that uptake was highest among the poorest groups.

Currently, there is a lack of information regarding equity in HIV testing at a national scale in Malawi. As such, our understanding of the likely barriers to achieving global goals in Malawi is incomplete. Out of the studies of HIV testing uptake that we could identify, none were carried out on a national sample, none studied inequity using standard tools such as the concentration index, and no study has yet explored the determinants of inequity in HIV testing in Malawi. There is a paucity of evidence on inequity in HIV testing in Malawi. Hence, this study aims to assess horizontal inequity in HIV testing at the national level in Malawi.

## Methods

This study calculates a decomposed concentration index (17) of access to HIV testing in Malawi. While the concentration index quantifies the extent of an inequality, or inequity, the decomposition method uses a regression-based approach to explore the determinants of inequity, that is, the contribution of different health need and non-need factors to the inequity identified in the concentration index. Common indicators of non-need variation in the literature include SES measures such as income and education (18). In this study, need factors include symptoms of sexually transmitted infections (STIs), while non-need factors include wealth, education, literacy and marital status. Here the ‘need factors’ reflect the need for health service use. Common indicators of need used in other studies include demographic variables, such as age and gender, and measures of health status (18). As the variable measuring uptake of HIV testing is binary (‘Have you ever been tested for HIV?’), a probit model is used in the regression (17).

A standard decomposition index (17) is not sufficient to measure horizontal inequity in HIV testing uptake in sub-Saharan African settings such as Malawi because of the complex relationship between HIV risk and SES. As mentioned earlier, in this context, wealthier groups have higher HIV prevalence (19, 20). This appears to contradict findings from other settings that poorer groups are more at risk of HIV (14, 19, 21). This may be a consequence of the fact that prevalence estimates are derived from testing outcomes, and access to testing may be skewed towards higher wealth groups (2). As such, a standard (pooled) concentration index for HIV testing is likely to non-randomly underestimate need and inequity among wealth groups because the method does not properly capture variation in need in different wealth groups (12).

This paper, therefore, applies the decomposition index method developed by Van de Poel et al. (12) in which the contribution of need variables in a decomposition index is divided into two parts: ‘corrected need’ and ‘discrimination’. This method for estimating corrected need is distinguished from traditional decomposition methods by the use of a reference group that is expected to realise vertical equity (12). Vertical equity implies individuals with different levels of needs are ‘appropriately’ consuming different amounts of health care (5). Van de Poel et al. (12) suggested that the highest wealth quintile is the reference group, while the pooled group is the whole population. Accordingly, this method captures variation in need and health service use between the reference and pooled groups – horizontal inequity – enabling us to extract the hidden vertical inequity that cannot be seen with conventional decomposition methods. Corrected need explores whether corrected, need-adjusted horizontal inequity is underestimated or not, given the pooled group. Discrimination explores how the health service use for a given need in a group compares with a reference group.

The detailed explanation of calculation of the concentration index, corrected need and horizontal inequity can be found in Appendix 1.

Wealth, as measured by an asset index, is included as a non-need variable in the decomposition approach. The decomposition analysis shows the contribution of each need and non-need factor to the pooled concentration index (CI) as shown in Equations 1 and 2 in Appendix 1. The concentration index depends on the relationship between the rank of SES and the health or other non-need variables, but not on the variation in the SES variable itself (17). When a socio-economic or wealth variable is included, as shown in Equation 4 in Appendix 1, the CI of wealth is calculated using the covariance between an individual's level of wealth and his/her wealth rank R_i_ (see Equation 1 in Appendix 1). Based on the given sample weight, individuals with the same level of wealth may have a different rank in DHS data (22). By definition, the CI of richer wealth quintiles is positive, while the CI of poorer quintiles is negative (12). In practice, when equity studies using decomposition analysis include the CI of ‘wealth’ in the non-need factors (12, 14, 17, 23), the focus is on the contribution of the wealth variable to the total CI, rather than on interpreting the CI of the wealth variable itself. For example, Wagstaff et al. (23) calculated a CI with the covariance between stunting and household consumption expenditure. In that study, the CI of household consumption expenditure was included as a non-need factor in the decomposition analysis. In this study of inequity in HIV testing in Malawi, once the CI of the ‘wealth’ variable is calculated, it is possible to estimate the contribution of wealth to the pooled CI because the contribution is the product of the elasticity and the CI of each variable.

Rural–urban inequality can be measured using a method similar to that described above. For the purposes of this study, a regional concentration index was calculated using the standard decomposition method, following steps in Equations 1–4 in Appendix 1. To compare rural and urban areas, a concentration index was calculated by estimating the covariance between each need variable, non-need variable and the wealth rank of people living in the area. A single asset index comprising five quintiles was developed for the whole of Malawi, without distinguishing between rural and urban areas (24).

To estimate the coefficients used in the decomposed concentration index, a probit model was used (17). A probit model allows the estimation of probabilities or marginal effects, imposing a normal distribution on the data (25). The mean of need variables and coefficients of the probit model were compared using a *t*-test for continuous variables and a chi-square test for categorical variables. All tests were conducted at the 95% confidence level. All analyses were carried out using Stata, Version 12 (StataCorp, College Station, TX, USA). Sample weights were applied when individuals were ranked by wealth.

### Data

DHSs are designed to collect national health and demographic data (24). Topics in the survey include fertility, contraception, breastfeeding, family planning, nutritional status of mothers and children, childhood illnesses and mortality, use of maternal and child health services, maternal mortality and domestic violence (6, 24). In addition, DHS 2004 and DHS 2010 in Malawi collected detailed HIV-related data including knowledge of and attitudes towards HIV/AIDS, receipt of an HIV test, HIV-related behavioural indicators, HIV status and symptoms of sexually transmitted diseases (STDs). The DHS in Malawi also tested a sub-sample of respondents for HIV. The age of the respondents ranges from 15 to 49 years for women and from 15 to 54 years for men (6).

This study uses data from two rounds of the DHS survey in Malawi: the 2004 round and the 2010 round. This enables calculation of within-year inequity and a comparison of inequity between each survey year. The 2004 data used in this study include 15,091 households, 11,698 women aged 15–49 years and 3,261 men aged 15–54 years. The 2010 data set includes 27,000 households, 24,000 women and 7,000 men. Both samples were drawn over 522 clusters: 458 in rural areas and 64 in urban areas (26). Malawi is divided into 10 districts in the DHS: Blantyre, Kasungu, Machinga, Mangochi, Mzimba, Salima, Thyolo, Zomba, Lilongwe, Mulanje and other districts. Based on the FAO classification (27), Lilongwe, Mzimba, Blantyre and Zomba were classified as urban areas in the DHS.

A probability sample, which is defined as one in which the units are selected randomly with known and non-zero probabilities, was used in the DHS data collection (28). Households were preselected in the central office before the start of data collection (28). Trained field staff conducted interviews only with the preselected households to avoid bias. Sample size was determined based on the calculation of sample size using relative standard error (RSE). Further details on the DHS sampling methodology can be found elsewhere (28).

The dependent variable in these analyses is ‘ever tested for HIV’. This takes the value of ‘1’ if the respondent has ever tested for HIV, and ‘0’ if they have never tested. Three questions on experience of STD symptoms in DHS 2010 and DHS 2004 are used as need variables in the analysis: 1) a diagnosed STD in the last 12 months, 2) a genital sore or ulcer in the last 12 months or 3) genital discharge in the last 12 months. A number of previous studies have used symptoms as need indicators in empirical analyses of equity (29, 30). The symptoms used in this study may be indicators of HIV infection (31), and patients should be referred for an HIV test when these symptoms are observed (31, 32). The presence of STIs also increases the possibility of transmitting HIV (8). The SES variables were selected as non-need variables including wealth as measured by an asset index, literacy, education and marital status.

Ethical approval was not required for this study because it was a secondary analysis of open-access data for which ethical approval had already been obtained.

## Results

### HIV testing uptake in 2010

Table 1 describes HIV testing uptake by SES in 2010 in Malawi. The data reveal significant differences in HIV testing by SES, especially among men. Three quarters (74.5%) of women and over half (53.7%) of all men reported that they have been tested for HIV. In terms of region, literacy, education, marriage and wealth, those who have been tested are significantly different from those who have not been tested (*p*<0.05). Testing is about 10 percentage points more common in the Northern region (79.2% among women and 61.6% among men) than in the Central region, and men in the Southern region are also lagging behind (52.3%). Literate women and men have been tested more often, but the gap is small for women and relatively large for men; 43.4% of illiterate men and 57.1% of men who can read a whole sentence have received an HIV test compared with 73.3 and 72.7% of women, respectively. The difference between primary and secondary education is relatively small for women (73.5% vs. 79.3%) but large for men (48.3% vs. 67.7%). The gap in HIV testing by wealth quintile is smaller than the gap by education: 72.1% of the poorest women and 49.7% of the poorest men have been tested, compared with 76% of the richest women and 59.9% of the richest men. There is very little difference between never-married (mostly young) women and men (40.2 and 42.5%, respectively). However, the difference between married and never-married women is much larger (43 percentage points) than the difference between married and never-married men (18 percentage points). Widowed and divorced women report lower levels of testing than women in a relationship (whether married, living together or not living together). Few men are divorced, widowed or not living together in this context. These results show a positive association between higher SES and the uptake of HIV testing.

**Table 1:** HIV testing by socio-economic status, Malawi DHS 2010

		Women (*N*=22,716)			Men (*N*=7,114)		
			
		Not tested (*N*=5,788) (% not tested)	Tested (*N*=16,928) (% tested)	*p*	Not tested (*N*=3,293) (% not tested)	Tested (*N*=3,821) (% tested)	*p*
Region	Northern	858 (20.8)	3,275 (79.2)	<0.001	491 (38.4)	789 (61.6)	<0.001
	Central	2,360 (30.4)	5,399 (69.6)		1,248 (48.4)	1,329 (51.6)	
	Southern	2,570 (23.7)	8,254 (76.3)		1,554 (47.7)	1,703 (52.3)	
Literacy	Cannot read at all	1,930 (26.7)	5,305 (73.3)	0.014	788 (56.6)	604 (43.4)	<0.001
	Able to read only parts of sentence	544 (25.6)	1,585 (74.4)		284 (52.2)	260 (47.8)	
	Able to read whole sentence	3,314 (24.8)	10,038 (75.2)		2,221 (42.9)	2,957 (57.1)	
Education	No education	912 (27.3)	2,431 (72.7)	<0.001	257 (58)	186 (42)	<0.001
	Primary	4,007 (26.5)	11,104 (73.5)		2,371 (51.7)	2,213 (48.3)	
	Secondary	817 (20.7)	3,125 (79.3)		608 (32.3)	1,275 (67.7)	
	Higher	52 (16.3)	268 (83.8)		57 (27.9)	147 (72.1)	
Marriage	Never married	2,676 (59.8)	1,801 (40.2)	<0.001	1,542 (57.5)	1,142 (42.5)	<0.001
	Married	2,212 (16.6)	11,099 (83.4)		1,407 (39.5)	2,158 (60.5)	
	Living together	306 (16)	1,612 (84)		239 (39.1)	373 (60.9)	
	Widowed	190 (22.4)	660 (77.6)		14 (48.3)	15 (51.7)	
	Divorced	244 (20.9)	924 (79.1)		53 (41.7)	74 (58.3)	
	Not living together	160 (16.1)	832 (83.9)		38 (39.2)	59 (60.8)	
Wealth	Poorest	1,248 (27.9)	3,229 (72.1)	0.001	568 (50.3)	562 (49.7)	<0.001
	Poorer	1,137 (25.6)	3,305 (74.4)		730 (50.6)	713 (49.4)	
	Middle	1,166 (25)	3,491 (75)		695 (47.5)	768 (52.5)	
	Richer	1,155 (24.9)	3,479 (75.1)		680 (44.4)	851 (55.6)	
	Richest	1,082 (24)	3,424 (76)		620 (40.1)	927 (59.9)	

*P*-value was calculated using chi-square test. No reply not included.

Table 2 presents the mean values and concentration indices for the need and non-need variables. Genital sore or ulcer is the most commonly reported of the three indicators of need (6.9% of women and 3.4% of men). Among women, the CI for each need variable is close to zero. Among men, the CI is small and negative, indicating that need is concentrated among the relatively poor. This result is surprising given that HIV prevalence is higher among the relatively wealthy.

**Table 2:** Descriptive summary of need and non-need variables and their concentration indices, Malawi DHS 2010

		Women (*N*=22,716)					Men (*N*=7,114)				
			
Variable		N	Mean	CI	SD	*p*^a^	N	Mean	CI	SD	*p*^a^
Test	Ever tested for HIV	22,716	0.745		0.436		7,114	0.537		0.499	
N1	Any STDs in last 12 months	377	0.016	0.003	0.127	<0.001	113	0.016	−0.034	0.124	0.1341
N2	Genital sore/ulcer in last 12 months	1,594	0.069	0.002	0.253	<0.001	245	0.034	−0.045	0.182	0.0062
N3	Genital discharge in last 12 months	860	0.037	0.002	0.189	<0.001	183	0.025	−0.006	0.156	0.9266
Wealth	Pooled	22,716	3.011	0.264	1.408	0.001	7,114	3.130	0.249	1.381	<0.001
	Lowest wealth quintile	4,477	0.197		0.398		1,130	0.159		0.366	
	Second lowest wealth quintile	4,442	0.196		0.397		1,443	0.203		0.402	
	Middle wealth quintile	4,657	0.205		0.404		1,463	0.206		0.404	
	Second upper wealth quintile	4,634	0.204		0.403		1,531	0.215		0.411	
	Upper wealth quintile	4,506	0.198		0.399		1,547	0.217		0.413	
Literacy	Pooled	22,716	1.269	0.127	0.913	0.014	7,114	1.532	0.075	0.800	<0.001
	Cannot read at all	7,235	0.318		0.466		1,392	0.196		0.397	
	Able to read only parts of sentence	2,129	0.094		0.291		544	0.076		0.266	
	Able to read whole sentence	13,352	0.588		0.492		5,178	0.728		0.445	
Education	Pooled	22,716	1.055	0.129	0.612	<0.001	7,114	1.260	0.116	0.612	<0.001
	No education	3,343	0.147		0.354		443	0.062		0.242	
	Primary	15,111	0.665		0.472		4,584	0.644		0.479	
	Secondary	3,942	0.174		0.379		1,883	0.265		0.441	
	Higher	320	0.014		0.118		204	0.029		0.167	
Marriage	Pooled	22,716	1.291	−0.076	1.228	<0.001	7,114	0.825	−0.044	0.910	<0.001
	Never married	4,477	0.197		0.398		2,684	0.377		0.485	
	Married	13,311	0.586		0.493		3,565	0.501		0.500	
	Living together	1,918	0.084		0.278		612	0.086		0.280	
	Widowed	850	0.037		0.190		29	0.004		0.064	
	Divorced	1,168	0.051		0.221		127	0.018		0.132	
	Not living together	992	0.044		0.204		97	0.014		0.116	

CI, confidence interval; SD, standard deviation; STDs, sexually transmitted diseases.

Variables test, N1, N2 and N3 take the value 1 for ‘yes’ and 0 for ‘no’.

a Calculated using *t*-test for need variables and chi-square test for non-need variables.

On the other hand, CIs for non-need factors were positive and far bigger than zero other than ‘marriage’.

Table 3 presents the results of decomposing the CI for HIV testing uptake in DHS 2004 and 2010 in Malawi. It also shows the contributions of the need and non-need variables to the estimated socio-economic inequity in HIV testing. The sum of the homogeneous contributions of the need variables from the standard decomposition is approximately zero for both women and men. The sum of the contributions of corrected need is also approximately zero for both women and men. This shows that non-need variables explain all of the existing inequity in HIV testing in 2010.

**Table 3:** Decomposition of the concentration index for HIV testing, Malawi DHS 2010 and 2004

	Women					Men				
		
Need	Coefficient	*p*^a^	Homogeneous	Corrected need	*p*^a^	Coefficient	*p*^a^	Homogeneous	Corrected need	*p*^a^
DHS 2010										
N1(had any STDs in last 12 months)	0.097	0.000	0.000	0.000	0.915	0.022	0.667	0.000	0.000	0.560
N2(had genital sore/ulcer in last 12 months)	0.065	0.000	0.000	0.000	0.900	0.077	0.028	−0.001	0.000	0.225
N3(had genital discharge in last 12 months)	0.037	0.029	0.000	0.000	0.931	−0.001	0.990	0.000	0.000	0.119
Sum of need contribution			0.000	0.000				−0.001	0.000	
Non-need										
Wealth	0.009	0.000	0.010			0.002	0.707	0.003		
Literacy	−0.001	0.723	0.000			0.023	0.007	0.005		
Education	0.056	0.000	0.010			0.150	0.000	0.041		
Marriage^c^	0.079	0.000	−0.002			0.081	0.000	−0.009		
Sum of non-need contribution			0.018					0.040		
Horizontal inequity (HI)			0.008	0.008				0.04	0.04	
DHS 2004										
N1(had any STDs in last 12 months)	0.067	0.074	−0.001	0.000	0.528	0.026	0.718	0.002	0.000	0.065
N2(had genital sore/ulcer in last 12 months)	−0.003	0.831	0.000	0.000	0.166	0.041	0.300	0.002	0.000	0.118
N3(had genital discharge in last 12 months)	0.042	0.052	−0.003	0.000	0.003	0.000	0.992	0.000	−0.001	0.229
Sum of need contribution			−0.004	0.000				0.004	−0.001	
Non-need										
Wealth	0.022	0.000	0.116			0.023	0.000	0.109		
Literacy	0.001	0.817	0.001			−0.012	0.213	−0.009		
Education	0.049	0.000	0.045			0.101	0.000	0.082		
Marriage^b^	0.023	0.000	−0.001			0.027	0.000	−0.012		
Sum of non-need contribution			0.161					0.17		
Horizontal inequity (HI)			0.152	0.152				0.185	0.186	

STDs, sexually transmitted diseases.

The contribution of homogeneous need corresponds to the first term in Equation 6. Corrected need corresponds to the second term in Equation 6. Horizontal inequity is calculated by subtracting the need contribution from the unstandardised concentration index.

a calculated using *t*-test comparing corrected need with zero

b calculated using not married (divorced, widowed, never married and not living together) and living together (married and living together).

Horizontal inequity among both women and men is positive (0.008 and 0.040, respectively), indicating that for a given need the relatively wealthy are more likely to access HIV testing in Malawi. However, the degree of horizontal inequity is small, especially for women. There is no difference between horizontal inequity and corrected need-adjusted inequity.

**Fig. 1:**
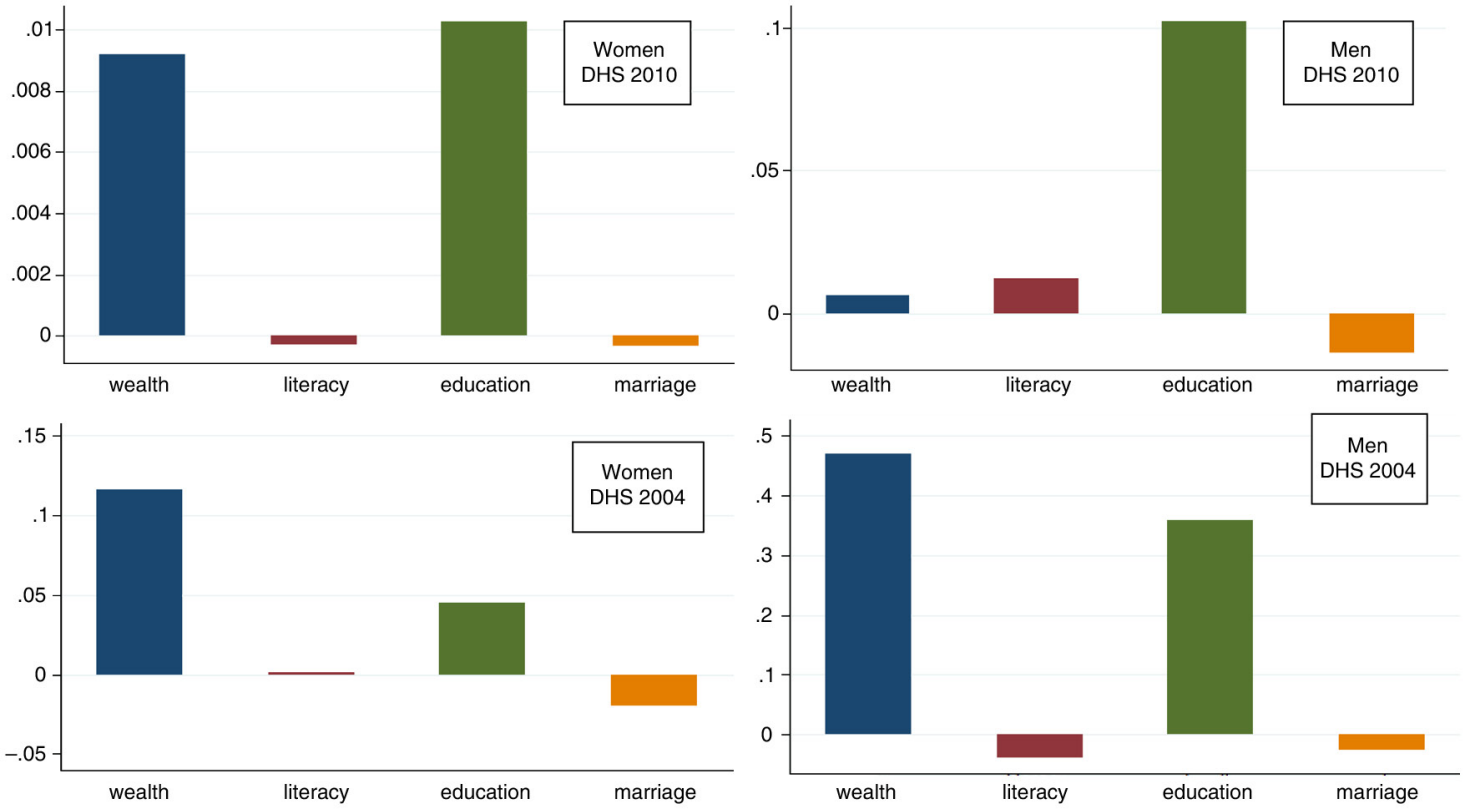
Contribution of non-need factors to the inequality in HIV testing uptake in men and women in Malawi (DHS 2010 and DHS 2004).

Figure 1 illustrates the contributions of the different non-need factors to the inequity in HIV testing. For both women and men in 2010, education was the most important non-need contributor to the concentration index. However, in 2004, wealth was a significant contributor for both women and men.

### Comparison of 2010 with 2004

The results from DHS 2010 contrast significantly with the data for 2004 in terms of access to HIV testing and inequity. Appendix 2 describes HIV testing by SES in 2004. Access to HIV testing dramatically increased between 2004 and 2010. In 2004, only 14.7% of women and 16.0% of men had been tested, compared with 74.5% of women and 53.7% of men in 2010. The pattern of socio-economic differences in 2004 was similar to that in 2010. However, one difference is worth noting. In 2004, socio-economic differences tended to be similar in magnitude among both women and men, while in 2010 the differences were more pronounced among men than among women. For example, the gap in testing between illiterate and fully literate decreased from 6 to 2 percentage points among women but increased from 7 to 14 percentage points among men. However, the gap in testing between married and never-married women was more pronounced in 2010 than that in 2004 (43 percentage points vs. 7 percentage points). The difference between the highest and the second highest wealth quintiles was relatively large in 2004 (8 and 11 percentage points among women and men, respectively) but relatively small in 2010 (1 and 4 percentage points, respectively).

The concentration indices for the need variables for women were negative but close to zero in 2004 (Appendix 3). On the other hand, for men, the CIs for the need variables were positive. This suggests that in 2004, need for HIV testing among men was concentrated among the relatively rich, but by 2010 the need for testing was equitably distributed.

Table 3 shows the decomposition of the CI for HIV testing in 2004. Horizontal inequity has fallen significantly between 2004 and 2010 from 0.152 to 0.008 for women and from 0.185 to 0.04 for men. In 2010, there was no difference between horizontal inequity and corrected need-adjusted inequity in 2004.

Figure 1 illustrates that, in 2004, wealth was the largest contributor to the concentration index for women and also greatly contributed to the same index for men. Although the extent of inequity in HIV testing fell between 2004 and 2010, the main contributor to inequity changed from wealth to education over this time, and this was the case for both men and women. In 2010, while wealth of men was given little weight, wealth of women was still of great importance.

### Decomposition analysis: rural–urban inequality

**Fig. 2:**
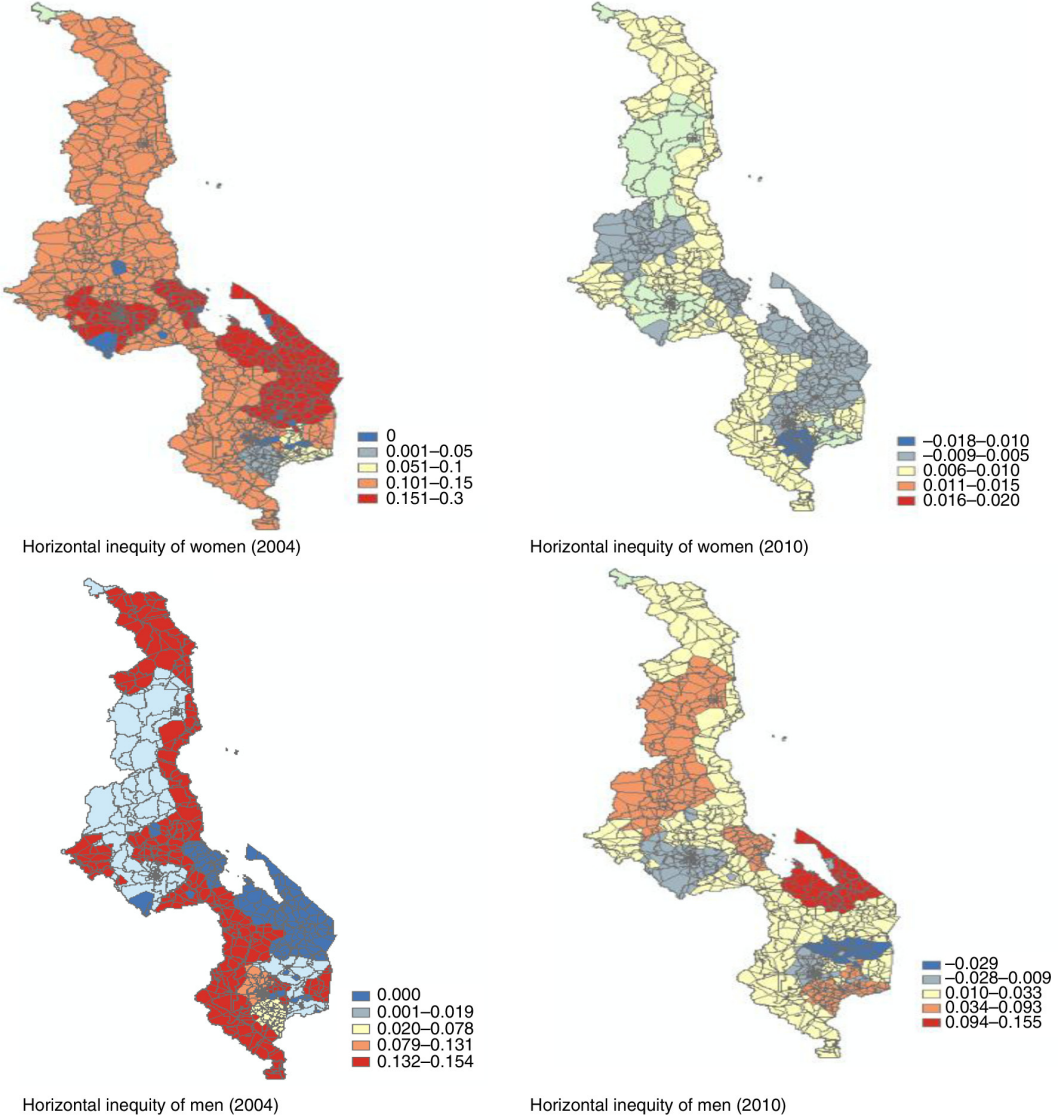
Map of horizontal inequity in Malawi. Horizontal inequity was calculated based on conventional concentration index.

Rural–urban inequality in HIV testing was also examined. The results showed that there exists little rural–urban inequality in HIV testing in 2010 (Table 4). In 2010, horizontal inequity among women living in rural areas was 0.005 compared with 0.014 among women living in urban areas, and 0.041 among men living in rural areas compared with 0.007 among men in urban areas. This means that access to HIV testing is more pro-rich among men in rural areas. In 2004, however, horizontal inequity among women and men living in urban areas was higher (0.18 and 0.211, respectively) than that among women and men living in rural areas (0.111 and 0.146, respectively). This result suggests that while access to HIV testing was affected by socio-economic factors in urban areas in 2004, men in rural areas were somewhat less affected by socio-economic factors in 2010. Fig. 2 illustrates regional variation in horizontal inequity in 2004 and 2010.

**Table 4:** Rural–urban inequality in HIV testing, Malawi DHS 2010 and 2004

Urban total (2010)				Rural total (2010)			
	
Women		Men		Women		Men	
			
	CI		CI		CI		CI
Wealth	0.218	Wealth	0.203	Wealth	0.268	Wealth	0.253
Literacy	0.113	Literacy	0.066	Literacy	0.123	Literacy	0.070
Education	0.145	Education	0.124	Education	0.115	Education	0.102
Marriage	−0.040	Marriage	−0.083	Marriage	−0.001	Marriage	−0.030
	Contribution				Contribution		
Need	−0.001	Need	0.002	Need	0.000	Need	−0.001
Non-need	0.012	Non-need	0.025	Non-need	0.007	Non-need	0.043
HI	0.014	HI	0.007	HI	0.005	HI	0.041
Urban total (2004)				Rural total (2004)			
	
Women		Men		Women		Men	
			
	CI		CI		CI		CI

Wealth	0.217	Wealth	0.206	Wealth	0.259	Wealth	0.237
Literacy	0.118	Literacy	0.086	Literacy	0.143	Literacy	0.072
Education	0.137	Education	0.111	Education	0.128	Education	0.103
Marriage	−0.035	Marriage	−0.062	Marriage	0.009	Marriage	−0.032
	Contribution				Contribution		
Need	−0.002	Need	0.000	Need	−0.003	Need	0.009
Non-need	0.178	Non-need	0.210	Non-need	0.105	Non-need	0.143
HI	0.180	HI	0.211	HI	0.111	HI	0.146

CI, concentration index; HI, horizontal inequity.

## Discussion

This study measures horizontal inequity in access to HIV testing in Malawi, using a decomposed concentration index. The approach of Van de Poel et al. (12) was applied to capture differences in need in Malawi. Rural–urban inequity was also examined using decomposition analysis. Inequity is explored using the 2010 Malawi DHS data to describe current access to HIV testing. This is compared with inequity calculated using the 2004 DHS data in order to show possible trends in access to HIV treatment.

Within the 2010 data, the need for HIV testing was equitably distributed, as reflected in equality between the standard index of horizontal inequity and corrected, need-adjusted inequity. In other words, the reference group of high-wealth men and women did not receive more HIV testing than the whole population. This finding may seem surprising, given that HIV prevalence is higher for higher SES groups in Malawi, as in the majority of sub-Saharan African countries (33). As described earlier, however, prevalence estimates are themselves affected by access to testing in a previous time period. Need in this statement refers to the need for testing, which does not suffer from the same bias. The variables we use to estimate this need – although potentially imperfect – are not subject to the same barriers to access that may affect estimates of HIV prevalence.

Comparing 2010 and 2004 data, the first notable observation is the total increase in access to HIV testing in the Malawian context. This increase in testing has also been accompanied by a significant reduction in horizontal inequity in HIV testing. These changes may in part be due to the significant financial support for HIV programmes in Malawi by global donors (34, 35). For instance, the Global Fund disbursed US$41 million for implementation of HIV treatment activities, including HIV testing, in 2005 (36). The number of HIV testing facilities as well as outreach programmes has increased, and national testing and counselling campaigns have been conducted (37). In 2008, a national programme offering HIV counselling and testing to 500,000 pregnant women was implemented at more than 500 sites (38). As a result, there has been a shift from facility-based testing to mobile and door-to-door testing, which appears to have had a net positive impact on testing access and also a positive impact on the equity of access to treatment – overcoming previous non-need barriers to HIV testing access (38).

Briefly, the strategies adopted for expanding access to HIV testing in Malawi have been successful in reducing inequity and expanding access. That reduction has taken place in both urban and rural areas. However, some degree of inequity remains among men living in rural areas, despite substantial investments in mobile clinics and door-to-door testing. A number of studies have found that distance is one of the biggest barriers to obtaining access to HIV testing and treatment in sub-Saharan Africa (39–41) and that transport costs constitute a substantial burden for patients in Malawi (33). In general, mobile testing is deemed a useful tool for offering HIV testing to low SES groups living in rural areas (16).

The reduced inequity observed in this study is of particular interest as global donors have been criticised for having a short-term results focus, with a need to attributed outcomes to their funding or support (42). Critics are concerned that programmes carried out by global health initiatives may create vertical service delivery structures that, to some extent, exacerbate health system problems (43). This seems not to have been the case in the Malawian context over the period from 2004 to 2010.

Given the findings of this analysis, regional decision-makers may want to focus on strategies or campaigns to improve gender equality in test uptake. While the stigma surrounding HIV infection and test uptake is well documented in this and other settings (44–46), women in Malawi appear to face a more significant risk of social sanction (10, 47). As men hold relatively more power within family structures and the Malawian social hierarchy more broadly, changing male perceptions of female testing will be critical to expanding access to HIV testing among Malawian women. One successful example of a campaign to reduce this gendered stigma surrounding HIV testing is the Malawi Radio Diaries programme (47). Malawi Radio Diaries featured HIV-positive male and female participants discussing their HIV status with one another. This programme helped change men's perception of women and HIV. Before the programme, many had thought that it was only promiscuous or low-status women who were at risk of contracting the disease (10, 47).

### Limitations

Although these findings advance our understanding of inequity in HIV testing uptake in Malawi and comparable contexts, the analysis has known limitations. Trends in the uptake of HIV testing since 2010 cannot be measured as more recent data are not yet available. The DHS Malawi 2014 was underway at the time of writing this paper. When DHS 2014 data become available, it will be possible to study whether the equity trends identified in this study have also continued beyond 2010. This is identified as a priority area for future study. Moreover, only three variables on STD symptoms were available within the DHS data sets. As a result, the need for HIV testing may be conservatively estimated in these analyses. The addition of further need variables in future analyses may enable a more nuanced analysis of inequity in HIV testing in the Malawian context.

## Conclusions

Measuring inequity in HIV testing uptake is important for improving access to care and informing health policy. While global stakeholders in HIV financing and care are embracing the 90–90–90 agenda, there has been a paucity of evidence on inequity in HIV testing uptake in local sites. This information can potentially highlight important barriers to care that may also constitute barriers to the attainment of these goals.

In resource-limited countries, expansion in access does not always result in improved equity in access. This study shows that access to HIV testing has significantly expanded in the Malawian context, and socio-economic inequity in HIV testing access has significantly reduced between 2004 and 2010. This may be attributed not only to increases in donor funding in this period but also to the strategies that donors used to expand testing access to the rural population. Nevertheless, it remains to be seen whether this observed low degree of inequity can be sustained as global priorities and funding patterns change.

The findings suggest that policymakers and policies should target lower SES groups, particularly rural men and women with low levels of education level. Strategies such as expanding mobile testing in rural areas and increasing awareness campaigns may be effective in expanding equitable access to HIV testing in Malawi. Finally, as education remains a contributor to horizontal inequity, the question of how to further increase testing among men and reduce the residual inequity among rural men in particular remains a priority area for further research.

## Paper context

*What is already known:* Socioeconomic status is a key determinant of HIV status in sub-Saharan African countries.

*What is new?* Access to HIV testing in Malawi significantly increased between 2004 and 2010. This increase has been accompanied by a concurrent reduction in horizontal inequity in access to HIV testing. Despite significant improvement, testing remains pro-rich among rural men. Education is a main contributor to horizontal inequity in HIV testing.

*What is the implication?* Malawi may serve as a model for the expansion of access in HIV testing. However, improving access among the most vulnerable in rural areas remains a policy challenge.

## Appendix 1: Calculation of the concentration index, corrected need and horizontal inequity

A concentration index is generally calculated as follows:

1 $$CI_{y}=\frac{2Cov(y_{i},R_{i})}{\mu }$$

where CI_y_ is the concentration index for health service use y, y_i_ is health service use for individual i, µ is the mean of health service use and R_i_ is individual i's fractional socio-economic rank.

We assume that health service use is determined by a set of k-independent variables (x_k_):

2 $$\text{y}=\alpha +\sum_{\text{k}}\beta_{\text{k}}{\text{x}}_{\text{k}}+µ$$

where β_k_ is a vector of coefficients and ɛ is the error term. Then the concentration index for health service use can be expressed as:

3 $$CI=\sum_{k}\frac{\beta_{k}\bar{x_{k}}}{\mu }C_{k}+GC/\mu$$

where ${\bar{\text{x}}}_{\text{k}}$ is the mean of the independent variable x_k_ and C_k_ is the concentration index of x_k_. GC_ɛ_ is the generalised concentration index for the error term, which is the remaining unexplained socio-economic inequality in the model (17).

Equation 3 consists of two parts: explained part and unexplained part. The explained part is made up of two elements: the concentration index for the independent variable (C_k_) and a measure of elasticity ($\frac{{\text{β}}_{\text{k}}{\bar{\text{x}}}_{\text{k}}}{\text{μ}}$). Elasticity ($\frac{{\text{β}}_{\text{k}}{\bar{\text{x}}}_{\text{k}}}{\text{μ}}$) is the impact of each independent variable on health service use. In other words, elasticity shows how much the dependent variable changes when one unit of the independent variable is changed. The CI in Equation 3 represents the extent to which the determinants of health service use are unequally distributed across wealth groups.

Keeping this in mind, the standard decomposition index including need and non-need variables is as follows:

4 $$\text{CI}=\sum_{\text{k}}\frac{\beta_{k}^{p}{\bar{x}}_{k}c_{k}}{\mu }+\sum_{m}\frac{r_{m}^{p}{\bar{z}}_{m}c_{m}}{\mu }+\text{G}{\text{C}}_{u}/\mu$$

where x_k_ and z_m_ are vectors of independent need and non-need variables, respectively. $\beta_{k}^{p}$ and $r_{m}^{p}$ are the regression coefficients of x_k_ and z_m,_ respectively, for the pooled group on health service use from Equation 2. ${\bar{\text{x}}}_{\text{k}}$ and ${\bar{\text{z}}}_{\text{m}}$ are the means of x_k_ and z_m,_ respectively. GC_u_ is again the generalised concentration index for the error term and µ is the mean of health service use. Conceptually, the first term on the right-hand side is the contribution of need variables (${\bar{\text{x}}}_{\text{k}}$) to the whole CI (‘need contribution’) and the second term is the contribution of non-need variables (${\bar{z}}_{m}$) to the whole CI (‘non-need contribution’), taking into account both the elasticity and the concentration index of the independent variables. This is an extension of Equation 3 above.

Jones and Ropez (48) then introduced another form of standard decomposition based on Equation 4 as follows:

5 $$\text{CI}=\sum_{\text{k}}\frac{\beta_{k}^{p}{\bar{x}}_{m}C_{k}}{\mu }+\frac{2}{\mu N}\sum_{k}\sum_{i}x_{ik}\left(\beta_{kg}-\beta_{k}^{p}\right)\left(R_{i}-\frac{1}{2}\right)+\sum_{\text{m}}\frac{r_{m}^{p}{\bar{z}}_{m}C_{m}}{\mu }+\frac{2}{\mu N}\sum_{m}\sum_{i}z_{m}\left(r_{mg}-r_{m}^{p}\right)\left(R_{i}-\frac{1}{2}\right)+\frac{2}{\mu }\mathrm{cov}\left(\alpha_{g},R_{i}\right)+\frac{2}{\mu }\mathrm{cov}\left(u_{i},R_{i}\right)$$

In this specification, the first and third terms are referred to as the homogeneous contributions, and the second and fourth terms are referred to as the heterogeneous contributions of need and non-need variables to the CI, respectively. The homogeneous contribution terms assume that the effects are the same across wealth groups. The second and fourth terms are the covariance between the regression coefficients and the socio-economic rank (R_i_) of individual i in wealth group g. These terms represent the heterogeneous contribution of the coefficient of the pooled values for need and non-need variables, respectively. The fifth term corresponds to the covariance between the fractional rank and group intercepts, and means the contribution of group differences in health service use to socio-economic status (SES)-associated inequality. The sixth term is the remaining unexplained inequality in health service use (48).

### Corrected need and horizontal inequity

Van de Poel et al. (12) split the second term in Equation 5 into two parts and labelled them ‘corrected need’ and ‘discrimination’, respectively. This method is distinguished from traditional decomposition methods by employing a reference group as a way of incorporating normative choice.

In this method, an asset index is used to split the population into wealth groups, and coefficient estimates from a pooled regression are compared with coefficient estimates from a regression using only the highest wealth group. The highest wealth group is expected to achieve higher levels of access in a use–need relationship (12). The ‘corrected need’ component of the CI can then be obtained after splitting the heterogeneous contributions of need and non-need variables into two parts: the corrected need effect and discrimination (12). The decomposition index can thus be disaggregated as follows:

6 $$\text{CI}=\sum_{\text{k}}\frac{\beta_{k}^{p}{\bar{x}}_{k}C_{k}}{\mu }+\frac{2}{\mu N}\sum_{i}\left(\beta_{kgr}-\beta_{k}^{p}\right)\sum_{i}x_{ik}\left(R_{i}-\frac{1}{2}\right)+\frac{2}{\mu N}\sum_{k}\sum_{i}x_{ik}\left(\beta_{kgr}-\beta_{kgr}\right)\left(R_{i}-\frac{1}{2}\right)+\sum_{m}\frac{r_{m}^{p}{\bar{z}}_{m}C_{m}}{\mu }+\frac{2}{\mu N}\sum_{m}\sum_{i}z_{m}\left(r_{mg}-r_{m}^{p}\right)\left(R_{i}-\frac{1}{2}\right)+\frac{2}{\mu }\mathrm{cov}\left(\alpha_{g},R_{i}\right)+\frac{2}{\mu }\mathrm{cov}\left(u_{i},R_{i}\right)$$

where $\beta_{k}^{p}$ and $r_{m}^{p}$ are the parameters from the original model (4). β_kgr_ is the coefficient from the reference (high-wealth) group. β_kg_ is the coefficient from a wealth quintile subgroup other than the reference group. The first and fourth terms are the homogeneous contributions from the standard decomposition and identical to the first and third terms in Equation 5, respectively. As explained previously, the second and third terms are referred to as ‘corrected need’ and ‘discrimination’, respectively. These terms are the main difference from Equation 5.

The second term of Equation 6 is the contribution of corrected need to the CI:

7 $$\frac{2}{\mu N}\sum_{k}\left(\beta_{kgr}-\beta_{k}^{p}\right)\sum_{i}x_{ik}\left(R_{i}-\frac{1}{2}\right)$$

where $\beta_{kgr}-\beta_{k}^{p}$ is the difference between the parameter estimates from the reference group and the pooled population regressions, respectively. x_ik_ is the need variable of individual i and µ is the mean of health service use. N is the total population and R_i_ is the fractional rank of individual i. Corrected need will be positive if the reference group uses more health services, and need is also concentrated more on this group. Likewise, corrected need will be negative if the highest wealth group uses more health services, but need is more concentrated on the poorest wealth group.

In general, unstandardised horizontal inequity (HI) is estimated by subtracting the contribution of need variables from the concentration index:

8 $$HI=C-\sum_{k=1}^{k}\frac{\beta_{k}^{p}{\bar{x}}_{k}C_{k}}{\mu }$$

So, higher the horizontal inequity is, the higher is the contribution of non-need variables to the concentration index.

Corrected need-adjusted horizontal inequity (12) is calculated by subtracting the contributions of both need and corrected need from CI:

9 $$HI=CI-\sum_{k=1}^{k}\frac{\beta_{k}^{p}{\bar{x}}_{k}C_{k}}{\mu }-\frac{2}{\mu N}\sum_{k}\left(\beta_{kgr}-\beta_{k}^{p}\right)\sum_{i}x_{ik}\left(R_{i}-\frac{1}{2}\right)$$

Horizontal inequity will be lower if corrected need is positive, and vice versa. We do not have to consider Van de Poel's discrimination term because discrimination is effectively captured on the right-hand side of Equation 9 as a result of the estimation of horizontal inequity, given Equation 6.

## Appendix 2: HIV testing by socio-economic status (DHS 2004)

**Table:**

		Women			Men		
			
		Not tested (*N*=9,692) (% not tested)	Tested (*N*=1,670) (% tested)	*p*	Not tested (*N*=2,696) (% not tested)	Tested (*N*=513) (% tested)	*p*
Region	Northern	1,308 (83)	268 (17)	<0.001	352 (78)	99 (22)	0.001
	Central	3,604 (89.3)	431 (10.7)		1,056 (85.2)	183 (14.8)	
	Southern	4,780 (83.1)	971 (16.9)		1,288 (84.8)	231 (15.2)	
Literacy	Cannot read at all	3,848 (88.7)	492 (11.3)	<0.001	604 (89.5)	71 (10.5)	<0.001
	Able to read only parts of sentence	833 (85.9)	137 (14.1)		157 (86.3)	25 (13.7)	
	Able to read whole sentence	5,011 (82.8)	1,041 (17.2)		1,935 (82.3)	417 (17.7)	
Education	No education	2,325 (89)	288 (11)	<0.001	323 (91)	32 (9)	<0.001
	Primary	6,121 (86.4)	960 (13.6)		1,793 (87.7)	251 (12.3)	
	Secondary	1,205 (75.3)	395 (24.7)		554 (73.7)	198 (26.3)	
	Higher	41 (60.3)	27 (39.7)		26 (44.8)	32 (55.2)	
Marriage	Never married	1,679 (91.1)	165 (8.9)	<0.001	882 (87.1)	131 (12.9)	0.057
	Married	6,438 (84.4)	1,188 (15.6)		1,700 (82.6)	358 (17.4)	
	Living together	447 (82.9)	92 (17.1)		29 (82.9)	6 (17.1)	
	Widowed	348 (85.7)	58 (14.3)		13 (86.7)	2 (13.3)	
	Divorced	487 (84.3)	91 (15.7)		36 (80)	9 (20)	
	Not living together	293 (79.4)	76 (20.6)		36 (83.7)	7 (16.3)	
Wealth	Poorest	1,773 (89.4)	211 (10.6)	<0.001	363 (87.9)	50 (12.1)	<0.001
	Poorer	1,984 (87.9)	273 (12.1)		602 (90.7)	62 (9.3)	
	Middle	2,145 (87.6)	305 (12.4)		632 (85.9)	104 (14.1)	
	Richer	2,032 (85.2)	353 (14.8)		614 (84.1)	116 (15.9)	
	Richest	1,758 (76.9)	528 (23.1)		485 (72.8)	181 (27.2)	

*P-*value was calculated using chi-square test. No reply was excluded.

## Appendix 3: Descriptive summary of need, non-need variables and their concentration indices (DHS 2004)

**Table:**

		Women (*N*=11,362)					Men (*N*=3,209)				
			
Variable		N	Mean	CI	SD	*p*^a^	N	Mean	CI	SD	*p*^a^
Test	Ever tested for HIV	11,362	0.147		0.354		3,209	0.160		0.367	
N1	Any STDs in last 12 months	106	0.009	−0.036	0.094	0.0058	25	0.008	0.217	0.088	0.2725
N2	Genital sore/ulcer in last 12 months	614	0.052	−0.033	0.223	0.3599	99	0.030	0.080	0.170	0.1884
N3	Genital discharge in last 12 months	346	0.029	−0.099	0.168	0.0168	64	0.019	0.093	0.137	0.7034
Wealth	Pooled	11,362		0.255		<0.001	3,209	0.130	0.235	0.336	<0.001
	Lowest wealth quintile	1,984	0.177		0.381		413	0.130		0.336	
	Second lowest wealth quintile	2,257	0.200		0.400		664	0.207		0.405	
	Middle wealth quintile	2,450	0.215		0.411		736	0.229		0.420	
	Second upper wealth quintile	2,385	0.209		0.406		730	0.227		0.419	
	Upper wealth quintile	2,286	0.199		0.400		666	0.207		0.405	
Literacy	Pooled	11,362	1.142	0.151	0.946	<0.001	3,209	1.519	0.079	0.821	<0.001
	Cannot read at all	4,340	0.387		0.487		675	0.212		0.409	
	Able to read only parts of sentence	970	0.085		0.279		182	0.057		0.232	
	Able to read whole sentence	6,052	0.528		0.499		2,352	0.731		0.444	
Education	Pooled	11,362	0.918	0.148	0.624	<0.001	3,209	1.158	0.113	0.625	<0.001
	No education	2,613	0.233		0.423		355	0.111		0.314	
	Primary	7,081	0.622		0.485		2,044	0.638		0.481	
	Secondary	1,600	0.139		0.346		752	0.233		0.423	
	Higher	68	0.006		0.077		58	0.018		0.133	
Marriage	Pooled	11,362	1.238	−0.099	1.128	<0.001	3,209	0.798	−0.045	0.803	0.057
	Never married	1,844	0.163		0.369		1,013	0.163		0.369	
	Married	7,626	0.671		0.470		2,058	0.671		0.470	
	Living together	539	0.047		0.212		35	0.047		0.212	
	Widowed	406	0.036		0.185		15	0.036		0.185	
	Divorced	578	0.051		0.219		45	0.051		0.219	
	Not living together	369	0.033		0.177		43	0.033		0.177	

Variables test, N1, N2 and N3 take the value 1 for ‘yes’ and 0 for ‘no’.

a Calculated using t-test comparing corrected need with zero.
